# Hematopoietic stem cell transplant with a carrier donor rescues CD40L deficiency

**DOI:** 10.70962/jhi.20250212

**Published:** 2026-02-17

**Authors:** Christen L. Ebens, Smitha Hosahalli Vasanna, Diana Vilkama, Tamara C. Pozos, Attila Kumánovics, Alison Hornyak, Amir A. Sadighi Akha

**Affiliations:** 1 Division of Blood and Marrow Transplant & Cellular Therapy, Department of Pediatrics, University of Minnesota, Minneapolis, MN, USA; 2 Department of Clinical Immunology, Children’s Minnesota, Minneapolis, MN, USA; 3 Department of Laboratory Medicine and Pathology, Mayo Clinic, Rochester, MN, USA; 4 Division of Infectious Disease, Department of Pediatrics, Sanford Health, Fargo, ND, USA

## Abstract

This study demonstrates successful hematopoietic stem cell transplant of CD40L deficiency using an asymptomatic carrier mother, leading to sustained 32% CD40L expression, normal recovery of T and B cell development and diversity, and expected growth and development 7.5 years post-transplant.

## Teaser

Ebens et al. demonstrate successful allogeneic hematopoietic cell transplant of CD40L deficiency using an asymptomatic carrier mother donor, with achievement of 32% CD40L expression, recovery of thymopoiesis, normal T cell diversity, and functional outcomes of T and B cell interactions.

## Introduction

CD40 ligand (CD40L) deficiency, or X-linked hyper IgM syndrome, is a combined deficiency of humoral and cellular immunity resulting from pathogenic variants in *CD40LG* on the X chromosome. The transmembrane glycoprotein CD40L expression on CD4 lymphocytes in germinal centers is required for co-stimulation of B cells and, in turn, immunoglobulin class switch recombination, somatic hypermutation, and development of memory B cells. In CD40L deficiency, patients develop dysgammaglobulinemia and absent/near absent class-switched memory B cells. CD40L engagement with the CD40 receptor on monocytes and dendritic cells is also important for T cell activation and development. The clinical phenotype of CD40L deficiency includes severe, recurrent, or recalcitrant sinopulmonary and/or gastrointestinal infections, including those caused by opportunistic pathogens such as *Pneumocystis jirovecii* and *Cryptosporidium parvum* (1).

Medical management of CD40L deficiency consists of immunoglobulin replacement, antimicrobial prophylaxis, and granulocyte-stimulating factor for severe neutropenia. The only curative option for CD40L deficiency is allogeneic hematopoietic cell transplantation (alloHSCT). In addition to alleviating infection risk and failure to thrive, successful alloHSCT avoids future development of autoimmunity and malignancy resulting from CD40L deficiency. A multinational report of 130 patients who underwent alloHSCT for CD40L deficiency showed superior overall survival when completed prior to age 2 years, with use of HLA-matched donors and myeloablative conditioning (2). Notably, achievement of >50% donor lymphocyte chimerism was associated with adequate B cell function to cease immunoglobulin replacement.

Use of a female carrier as a donor for CD40L deficiency poses unique questions, including stability of lyonization, threshold of CD40L expression, and ability of partial correction to support normal B cell differentiation, immunoglobulin production, vaccine response, and T cell repertoire/function. Chandrakasan et al. (3) reported a case series of four patients with CD40L deficiency transplanted with carrier stem cells, demonstrating successful engraftment with CD40L expression near that of the donor (range 37–78.2%), no opportunistic infections or cases of graft-versus-host disease, and independence from immunoglobulin replacement in three of four. We present an additional case of carrier donor alloHSCT for CD40L deficiency, including measures of neothymopoiesis, T cell repertoire diversity, and lymphocyte proliferation responses.

## Case presentation

A male infant presented to a community hospital at the age of 5 mo with severe viral pneumonia (non-SARS-CoV2 coronavirus, human metapneumovirus, and rhino/enterovirus) requiring mechanical ventilation. Following recovery, he was monitored with notable failure to thrive but no further immune evaluation. At 9 mo of age, he developed rhino/enteroviral pneumonia, requiring hospitalization and prolonged oxygen support. 2 days into hospitalization, he was transferred to pediatric infectious disease care at a regional hospital, where a comprehensive family history uncovered a maternal great-uncle who experienced recurrent infections and a maternal uncle with chronic cytomegalovirus infection and learning disabilities. With growing concern for an immunodeficiency, immunophenotyping was sent, revealing hypogammaglobulinemia (IgG <109, normal 232–1,411 mg/dl; IgM 67, normal 0–145 mg/dl), absent class-switched memory B cells, and absent CD40L expression and CD40-binding capacity. IVIG supplementation and trimethoprim-sulfamethoxazole *P. jirovecii* pneumonia (PJP) prophylaxis were initiated. With genetic testing for CD40L deficiency pending, the patient was transferred to a regional Children’s Hospital with an immunology service. Recurrent oxygen need during transport prompted a diagnostic bronchoscopy and alveolar lavage on the second day of admission, identifying PJP (then 10 mo of age). Trimethoprim-sulfamethoxazole was transitioned to intravenous treatment dosing, and steroids were added. With this higher dosing, the patient developed neutropenia that normalized with granulocyte colony-stimulating factor, requiring two doses over PJP treatment. Sanger sequencing of *CD40LG* (transcript NM_000074.2 at Prevention Genetics) returned with a hemizygous pathogenic variant in *CD40LG* c.661C>T (p.Gln221*).

## AlloHSCT course

With a diagnosis of CD40L deficiency, failure to thrive, and life-threatening infections, he was referred for alloHSCT. HLA typing was completed, and in the absence of a matched sibling donor, a fully matched unrelated donor was arranged. Unfortunately, the donor became ineligible due to a medical illness. The patient’s best immediately available donor option was his 11/12 (nonpermissive HLA-DPB1 mismatch) HLA-matched mother. She was heterozygous for the same *CD40LG* pathogenic variant but asymptomatic, with CD40L expression of 44%. At 1 year of age, this patient underwent reduced toxicity myeloablative conditioning with alemtuzumab (0.3 mg/kg daily × 3 from day −12), busulfan (dosed every 6 h intravenously with pharmacokinetic monitoring and dose adjustment to achieve a target cumulative area under the curve of 72 mg*h/L, days −9 to −6), and fludarabine (1.33 mg/kg daily from day −5 to −2), followed by unmanipulated bone marrow (total nucleated cells 6.91 × 10^8^/kg and CD34^+^ 6.7 × 10^6^/kg). Graft-versus-host disease prophylaxis included cyclosporine (6 mo) and mycophenolate mofetil (1 mo).

**Figure 1.**
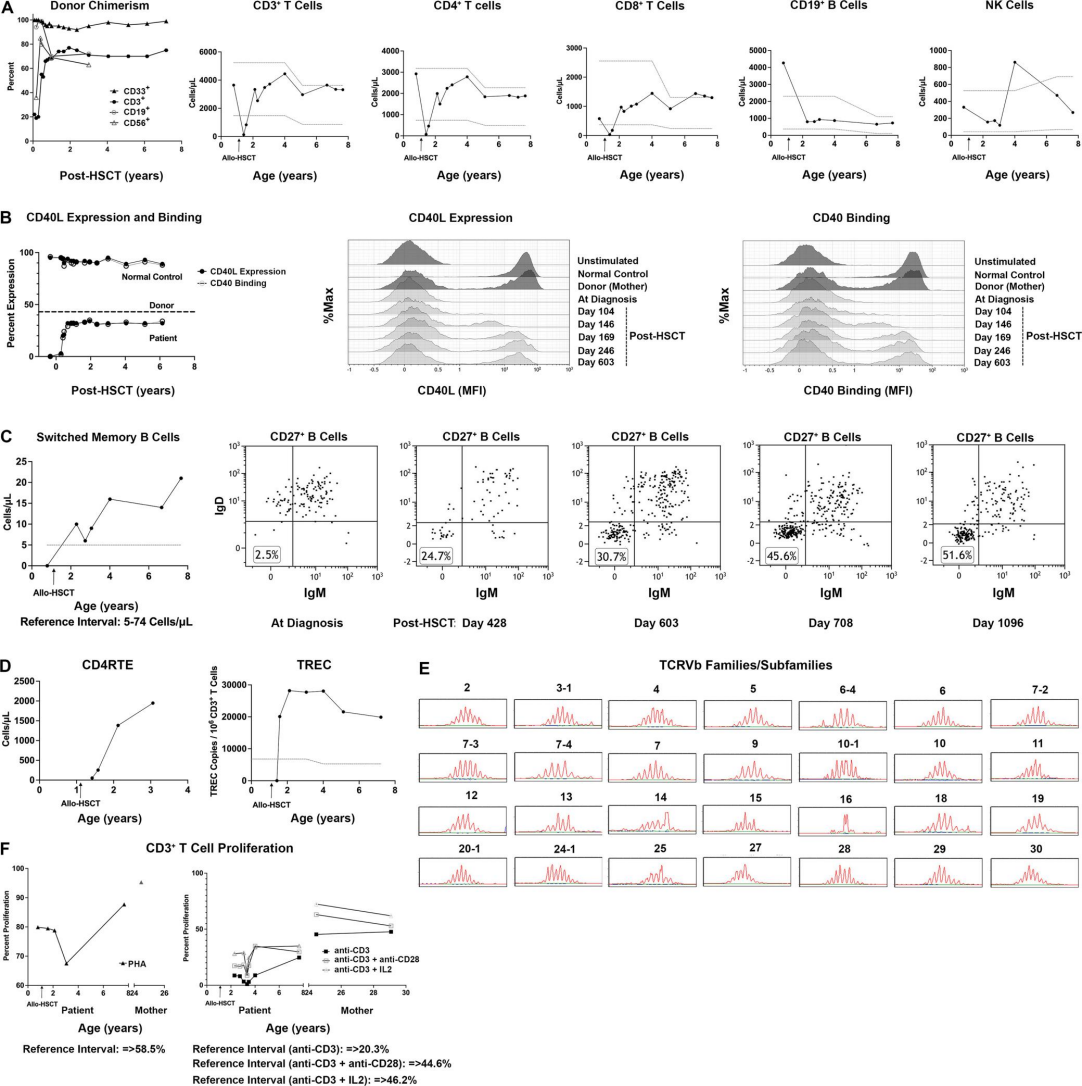
**Immune landscape and donor chimerism from diagnosis through alloHSCT. (A)** Monitoring of donor chimerism and enumeration of the patient’s CD3^+^ T cells, CD4^+^ T cells, CD8^+^ T cells, CD19^+^ B cells, and NK cells during the course of observation. The dotted gray lines define the upper and lower ends of the reference intervals, which were established during the validation of these clinical assays at Mayo Clinic. **(B)** Longitudinal monitoring of the patient’s CD40L expression and binding in comparison to the donor (left panel), with representative examples of the flow plots displayed in the middle (CD40L expression) and right (CD40 binding) panels. **(C)** Longitudinal enumeration of the absolute number of CD27+IgM-IgD class-switched memory B cells (left panel), with flow plots displaying their gradual increase as a proportion (%) of CD27^+^ memory B cells over time (subsequent panels). **(D)** Evaluating thymic output by quantifying CD4 recent thymic emigrants and T cell receptor excision circles. Values above the dotted gray line in the TREC plot are within the normal reference interval. **(E)** Evaluation of the patient’s TCRVβ repertoire. **(F)** Longitudinal assessment of the T cell proliferative response to PHA, anti-CD3, anti-CD3 + anti-CD28, and anti-CD3 + IL2. PHA, phytohemagglutinin.

The patient achieved neutrophil recovery on day +10 (absolute neutrophil count >500/mcl × 3 days) and platelet recovery on day +16 (platelet count of >20,000 × 3 days). His first 100 days post-alloHSCT were complicated by several infections, which resolved with appropriate therapy (rotaviral gastroenteritis and enterococcal, *Pseudomonas aeruginosa*, and *Streptococcus mitis* bacteremia). Major life-threatening transplant-associated complications such as graft-versus-host disease, venoocclusive disease of the liver, sepsis, or viremia were not observed. Peripheral blood chimerism revealed complete donor CD33^+^ and stable mixed donor CD3^+^, CD19^+^, and NK cell chimerism in the 60–70% range from +1 to +6 years post-alloHSCT (Fig. 1 A, left panel). He had sustained reconstitution of CD3^+^ T cells and CD4^+^ and CD8^+^ subsets, as well as CD19^+^ B cells and NK cells (Fig. 1 A). His day +104 CD40L expression and CD40 binding were extremely low at 3 and 2%, respectively, but following cyclosporine taper from 4 to 6 mo post-alloHSCT, they improved to the 20% range at +6 mo and stabilized in the low 30% range from +9 mo to present (+7.5 years; Fig. 1 B). The improvement in CD40L expression and binding coincided with a gradual normalization of switched memory B cell numbers and their proportion within the memory B cell compartment (Fig. 1 C).

Thymic output, quantified by T cell receptor excision circle and CD4^+^ recent thymic emigrants (CD4^+^CD45RA^+^CD31^+^) analysis, has been normal for age since +6 mo post-alloHSCT (Fig. 1 D). Additionally, at 6.5 years post-alloHSCT, TCRVβ spectratyping showed normal T cell receptor diversity (Fig. 1 E). Functionally, while T cell proliferative response to phytohemagglutinin was normal at +6 mo post-alloHSCT, the proliferative responses to anti-CD3 alone or in combination with anti-CD28 or soluble IL2 were not (Fig. 1 F). In theory, this could have been a cell-intrinsic effect of the pathogenic variant or a consequence of alloHSCT. As the donor has normal proliferative responses to anti-CD3 and the patient’s responses have eventually improved, it can be attributed to the latter. IgG replacement was discontinued at +2 years post-alloHSCT once IgM normalized, and all routine childhood immunizations began at +2.5 years (including live-attenuated vaccines, which were well tolerated). At +3 years post-alloHSCT, when the patient had normal numbers of lymphocytes, including normal class-switched B cells and adequate antibody production in response to polysaccharide-protein-conjugated immunizations (but low to polysaccharide immunizations), antifungal and anti-PJP prophylaxis were discontinued. Knowing polysaccharide immunization responses are T cell-independent and often decreased in young children, additional doses of pneumococcal vaccine were provided. Since peri-transplant recovery and now 7.5 years post-alloHSCT, this patient has experienced no life-threatening infections, autoimmunity/malignancy, and is demonstrating excellent growth and development, with symmetric weight and height around the 15–20th percentile for age.

### Conclusion

This study adds to the sparse literature on the subject. Consistent with the small prior series (3), post-alloHSCT CD40L expression/activity in the recipient was limited to that of his carrier mother and stable over time, giving further credence to the notion that partial restoration of CD40L expression and binding can reverse the CD40L deficiency phenotype. CD40L deficiency appears to be clinically cured in our patient with only 32% CD40L expression and CD40 binding, herein demonstrated to provide adequate co-stimulation for normal development of class-switched B cells. Additionally, our study provides a detailed analysis of successful thymus-derived T cell reconstitution and generation of a diverse T cell repertoire using a carrier donor in this combined immunodeficiency. With 7.5 years of post-alloHSCT follow-up and surveillance, these laboratory and clinical findings appear robust and stable.

Although rarely described in CD40L deficiency, skewed lyonization can result in symptomatic disease in a carrier female (4). While equivalently HLA-matched related donors convey decreased GvHD risk compared to unrelated donors, the risk of CD40L deficiency recurrence with the use of a carrier donor is a concern. With existing data on expression stability and successful use of carrier donors in CD40L deficiency here and in the literature (3), we would consider selection of a carrier donor over an unrelated donor, all other factors being equal (HLA match, ABO type, and CMV serostatus). However, we would limit the use of an asymptomatic individual with activated CD4 T cell protein expression at or exceeding 40% to offset the impact of mixed donor lymphoid chimerism. In addition to supporting the use of carrier donors in alloHSCT, this clinical and biologic threshold can inform goals for autologous gene therapies in development (5) and make CD40L deficiency an attractive target.

## Human subjects research

Parents of the human subject provided informed consent for treatment on a University of Minnesota institutional review board-approved study, MT2012-10C Arm B (listed at https://clinicaltrials.gov as NCT01652092), with data collection on a separate institutional review board-approved BMT Database study, in accordance with the Declaration of Helsinki.

## Data Availability

All data are available in the article.
